# Development and Validation of a Novel DNA Methylation-Driven Gene Based Molecular Classification and Predictive Model for Overall Survival and Immunotherapy Response in Patients With Glioblastoma: A Multiomic Analysis

**DOI:** 10.3389/fcell.2020.576996

**Published:** 2020-09-03

**Authors:** Zihao Wang, Lu Gao, Xiaopeng Guo, Wei Lian, Kan Deng, Bing Xing

**Affiliations:** Department of Neurosurgery, Peking Union Medical College Hospital, Chinese Academy of Medical Sciences and Peking Union Medical College, Beijing, China

**Keywords:** DNA methylation-driven genes, glioblastoma, GSEA, prognostic model, multiomic analysis

## Abstract

**Purpose:**

Glioblastoma (GBM) is the most common primary malignant tumor of the central nervous system, with a 5-year overall survival (OS) rate of only 5.6%. This study aimed to develop a novel DNA methylation-driven gene (MDG)-based molecular classification and risk model for individualized prognosis prediction for GBM patients.

**Methods:**

The DNA methylation profiles (458 samples) and gene expression profiles (376 samples) of patients were enrolled to identify MDGs using the MethylMix algorithm. Unsupervised consensus clustering was performed to develop the MDG-based molecular classification. By performing the univariate, least absolute shrinkage and selection operator (LASSO), and multivariate Cox regression analysis, a MDG-based prognostic model was developed and validated. Then, Bisulfite Amplicon Sequencing (BSAS) and quantitative real-time polymerase chain reaction (qPCR) were performed to verify the methylation and expressions of MDGs in GBM cell lines.

**Results:**

A total of 199 MDGs were identified, the expression patterns of which enabled TCGA and CGGA GBM patients to be divided into 2 clusters by unsupervised consensus clustering. Cluster 1 patients commonly exhibited a poor prognosis, were older in age, and were more sensitive to immunotherapies. Then, six MDGs (ANKRD10, BMP2, LOXL1, RPL39L, TMEM52, and VILL) were further selected to construct the prognostic risk score model, which was validated in the CGGA cohort. Kaplan-Meier survival analysis demonstrated that high-risk patients had significantly poorer OS than low-risk patients (logrank *P* = 3.338 × 10-6). Then, a prognostic nomogram was constructed and validated. Calibration plots, receiver operating characteristic curves, and decision curve analysis indicated excellent predictive performance for the nomogram in both the TCGA training and CGGA validation cohorts. Finally, *in vitro* BSAS and qPCR analysis validated that the expressions of the MDGs were negatively regulated by methylations of target genes, especially promoter region methylation.

**Conclusion:**

The MDG-based prognostic model could serve as a promising prognostic indicator and potential therapeutic target to facilitate individualized survival prediction and better treatment options for GBM patients.

## Introduction

Glioblastoma, corresponding to grade IV, is the most common (47.7%) primary malignant tumor of the central nervous system, with an incidence rate of 3.21 per 100,000 population according to the CBTRUS Statistical Report in the United States in 2011–2015 (Ostrom et al., 2018). GBM is the most aggressive and infiltrative brain tumor, with a 5-year OS rate of only 5.6% post-diagnosis (Ostrom et al., 2018). The standard treatment strategy for GBM is maximal safe surgical resection, followed by concurrent radiation and chemotherapy (Weller et al., 2014). However, despite the great progress made in GBM treatment, GBM still exhibits significant morbidity and mortality. With the rapid development of large-scale genome−sequencing technologies, numerous molecular biomarkers for GBM have been reported in the literature, including IDH 1/2 mutation, MGMT promoter methylation status, TERT promoter mutations, B-Raf proto-oncogene (BRAF) mutations, ATRX mutations, EGFR mutations, and 1p/19q codeletion (Smith et al., 2000; Liu et al., 2012; Chan et al., 2015; Bell et al., 2018). However, those biomarkers only present limited values in predicting survival of GBM patients in clinical applications. Thus, it is indispensable to explore the underlying molecular mechanisms and investigate prognostic biomarkers and therapeutic targets for GBM.

Epigenetic modifications have been reported to play an important role in the development and progression of multiple cancers (Wilting and Dannenberg, 2012). DNA methylation, one of the major components of epigenetic modification, is a crucial signaling tool that modulates genomic functions, especially regulation of the expression of oncogenes and tumor suppressor genes (Wilting and Dannenberg, 2012). Aberrant DNA methylation of the MDGs, including hypomethylation of oncogenes and hypermethylation of tumor suppressors, is a crucial process contributing to the oncogenesis and progression of multiple cancers, especially GBM (Kulis and Esteller, 2010; Wilting and Dannenberg, 2012; Aoki and Natsume, 2019). Recent studies have investigated various DNA methylation events in the pathogenesis, recurrence, and drug resistance of GBM (Klughammer et al., 2018; Aoki and Natsume, 2019). DNA methylation was also reported to provide a new option for early diagnosis and treatment of GBM (Gusyatiner and Hegi, 2018; Klughammer et al., 2018; Aoki and Natsume, 2019). However, previous studies mainly focused on single DNA methylation events, whereas global DNA methylation patterns with multiple MDGs have not been developed before for GBM. Global DNA methylation patterns may become a novel standard, replacing the conventional World Health Organization (WHO) grading system based on histological diagnosis due to its notable value in early diagnosis, subgroup classification, risk stratification, and prognosis prediction for GBM (Gusyatiner and Hegi, 2018; Klughammer et al., 2018; Aoki and Natsume, 2019).

In the present study, by performing a combined multiomic analysis based on transcriptomic and DNA methylation patterns, we first identified the aberrantly methylated and differentially expressed DNA MDGs by using the MethylMix algorithm. We developed and validated a novel MDG-based molecular classification of GBM, which was associated with prognosis and immunotherapy response. Then, an MDG-based risk score model was constructed and validated to predict the prognosis and serve as potential therapeutic targets for GBM. Finally, a novel promising prognostic nomogram with favorable predictive performance was constructed and validated based on the MDG signature and multiple clinicopathological parameters. Finally, *in vitro* BSAS and qPCR analysis were performed to validate the associations between promoter region methylations and expressions of the MDGs in GBM cell lines. Our study aimed to facilitate individualized survival prediction and better treatment options for both physicians and GBM patients according to the DNA methylation and transcriptomic patterns constructed in this study.

## Materials and Methods

### Data Acquisition and Processing

The DNA methylation data, level-three RNA sequencing data and corresponding clinical information of primary GBM patients were downloaded from TCGA^1^. The DNA methylation profiles included profiles of 10 normal and 448 tumor samples, and the gene expression profiles included profiles of 5 normal and 155 tumor samples. One hundred thirty-five samples were represented in both the DNA methylation data and the paired RNA sequencing data. The level-three RNA sequencing data and corresponding clinical information of the validation cohort, which included 216 GBM patients, were downloaded from the CGGA^2^ database. All patients without prognostic information were excluded. Ethics committee approval for this study was not necessary because the data were obtained from the TCGA and CGGA.

### Identification of Differentially Expressed Genes in GBM

The DEGs between GBM and normal samples of TCGA were screened using edgeR in R 3.5.1 (Robinson et al., 2010). Adjusted *P* (adj. P) values were calculated using the default Benjamini-Hochberg FDR method to reduce the false-positive rate. Adj. *P* < 0.01 and |Log_2_[fold change (FC)]| > 1 were considered as the cutoff criteria for identifying DEGs (Wang et al., 2019).

### Identification of DNA Methylation-Driven Genes and Enrichment Analyses

The MethylMix package in R 3.5.1 was used to perform a comprehensive analysis integrating DNA methylation data and gene expression data (Cedoz et al., 2018). First, the aberrantly methylated genes, which were based on the DEGs, between 448 GBM and 10 normal samples were screened by the LIMMA package (Wettenhall and Smyth, 2004). Second, the correlations between the methylation data and paired gene expression data of 135 GBM patients were assessed. The genes with a correlation coefficient <−0.3 and *P* value < 0.05 were chosen for further analysis. Then, the β mixture models were constructed to determine the disease-specific methylation status of multiple genes. Finally, considering the large disparity in sample size between the normal and tumor groups, independent sample *t*-test was performed and *P* < 0.05 were used to determine the MDGs. The aberrantly methylated and differentially expressed MDGs were then determined by the above-mentioned 4 steps (Cedoz et al., 2018).

The DAVID^3^ was employed to perform functional and pathway enrichment analysis of the MDGs (Huang et al., 2009). Gene ontology analysis, including analyses of the BP, CC and MF categories, was used for functional annotation, and KEGG analysis was used for pathway enrichment analysis (Ashburner et al., 2000; Kanehisa et al., 2017). A *P* value < 0.05 was considered statistically significant.

### Unsupervised Consensus Clustering of GBM Patients Based on the MDGs

Unsupervised consensus clustering, a k-means machine learning algorithm, was applied to explore a novel molecular classification of GBM patients based on the expression patterns of the MDGs using the “ConsensusClusterPlus” package. The clustering procedure was performed with 1000 iterations by sampling 80% of the data in each iteration. The optimal number of clusters was determined by the relative change in the area under the CDF curves of the consensus score and consensus heatmap. Then, the cluster quality measures called the IGP were applied to verify the similarities between different clusters in other independent datasets by using the “clusterRepro” package. Next, K-M survival analysis was performed to evaluate the prognosis of patients in different clusters. Comparisons of the clinicopathological variables between clusters were also performed to explore the associations between the MDG-based molecular classification and clinical features of GBM patients.

### Prediction of the Immunotherapy Responses of GBM Patients

The TIDE^4^ model is a computational method that integrates the expression signatures of T cell dysfunction and T cell exclusion to model tumor immune evasion (Jiang et al., 2018). The clinical response of ICB was predicted by the TIDE algorithm based on pretreatment tumor profiles. Then, an unsupervised method (SubMap^5^) was applied to predict the ICB response of the GBM patients in different MDG-based molecular subgroups (Hoshida et al., 2007).

### Construction and Validation of the MDG-Based Prognostic Risk Score Model

Univariate Cox regression analysis was performed to identify the associations between the expression of MDGs and patient OS in the TCGA training cohort. Prognosis-related genes with a *P* value < 0.05 were further screened by the LASSO and multivariate Cox regression analysis. Then, the prognostic risk score model based on the MDGs was constructed to predict patient OS. The risk score model was established with the following formula: risk score = Exp (Gene_1_) × β_1_ + Exp (Gene_2_) × β_2_ + ⋯ + Exp (Gene_*n*_) × β_*n*_, where Exp represents the expression level of the gene, and β represents the regression coefficient of each MDG calculated by the multivariate Cox regression analysis (Wang et al., 2019). A prognostic risk score for each patient was calculated according to the formula. The TCGA GBM patients were stratified into low-risk (low risk score) and high-risk (high risk score) groups according to the median value of their risk scores. Then, K-M survival curve analysis using the survival package was performed to estimate the prognosis of patients with high and low risk scores, and the survival differences between the high-risk and low-risk patients were evaluated by the two-sided log-rank test. The predictive accuracy/prognostic performance of the MDG-based prognostic model within 0.5, 1 and 3 years was evaluated by the Harrell’s C-index and time-dependent ROC curve analysis with the survcomp and survivalROC packages in R (Harrell et al., 1996; Alba et al., 2017). Both the C-index and AUC range from 0.5 to 1, with 1 indicating perfect discrimination and 0.5 indicating no discrimination. The prognostic model constructed by the TCGA training cohort was then validated in the CGGA GBM cohort in a similar manner.

Univariate and multivariate Cox regression analyses were performed with the TCGA training set and CGGA validation set, respectively, to determine whether the predictive power of the MDG-based prognostic risk score model was independent of other clinicopathological variables, including age, sex, new event, KPS, pharmacotherapy, radiotherapy, surgery, IDH mutation status, MGMT promoter status, TERT mutation status, BRAF mutation status, ATRX mutation status, EGFR mutation status, and 1p/19q status.

### Construction and Validation of the Prognostic Nomogram With the MDG Signature

All of the independent prognostic factors identified by the univariate and subsequent multivariate Cox regression analysis were used to establish a prognostic nomogram to evaluate the probability of 0. 5-, 1−, and 3−year survival for TCGA GBM patients using the rms package^6^ in R (Qian et al., 2018). The Schoenfeld Residuals Test was used to investigate the independence of residuals and time, and thereby to test the proportional hazard (PH) assumption in the Cox model. Only *P* > 0.05 can satisfy the PH assumption and the results of the Cox regression model are meaningful and reliable. The discrimination performance of the nomogram regarding prognosis was quantitatively assessed by the C-index and ROC curve analysis (Harrell et al., 1996). Calibration plots at 0.5, 1, and 3 years were also employed to visually evaluate the discriminative ability of the nomogram (Alba et al., 2017). In addition, DCA was performed to determine the clinical usefulness of the nomogram by quantifying the net benefits at different threshold probabilities in the GBM patients (Goeman, 2010). The best prediction model commonly has a high net benefit as calculated within the favorable probability. The prognostic nomogram was externally validated in the CGGA GBM cohort. All analyses were conducted using R version 3.5.1, and a *P* value < 0.05 was considered statistically significant. HRs and 95% CIs are reported where appropriate.

### Integrated Survival Analyses Based on the Expression and Methylation of MDGs

According to the median values of gene expression and DNA methylation level of MDGs, patients were divided into high-expression and low-expression groups and into high-methylation and low-methylation groups. Then, K-M survival analyses were performed to evaluate the associations between the expression levels or DNA methylation levels of the prognosis-related MDGs and OS. In addition, we performed integrated survival analyses based on the gene expression and methylation levels of MDGs to assess the survival differences between low-expression patients with high methylation and high-expression patients with low methylation.

### Gene Set Enrichment Analysis

Setting the expression level of a single gene as the population phenotype, GSEA^7^ was performed to identify related pathways and molecular mechanisms of the MDGs enrolled in the prognostic model of GBM patients (Subramanian et al., 2005). A nominal *P* value < 0.05 of the enrichment gene sets was considered statistically significant.

### Cell Culture and 5-aza-2′-Deoxycytidine (DAC) Treatment

The GBM cell line U251 was preserved in our institute (Chinese Academy of Medical Sciences and Peking Union Medical College, Beijing, China) and was cultured in (MEM, Gibco) supplemented with 10% fetal bovine serum and 1% penicillin/streptomycin. U251 cells in culture were treated with 5 μM/L DAC (Sigma-Aldrich) for 120 h, and the medium was changed every day due to its instability. For experiments involving DAC treatment, DMSO was utilized as the control treatment. The cells were harvested (120 h after starting the culture) for extraction of genomic DNA and total RNA for analysis of DNA methylation and gene expression.

### Bisulfite Amplicon Sequencing

Genomic DNA was prepared by the proteinase K method. BSAS was performed as described in the literature (Masser et al., 2015). Prediction of CpG islands in promoter regions and design of BSAS primers were performed using MethPrimer 2.0 software^8^ according to the genomic sequence around the TSS. Three pairs of primers were predicted and selected for each gene to perform BSAS. The BSAS primers are listed in Supplementary Table 1. The site relative to TSS and the corresponding chromosomal location of the CpGs located in the promoter region are listed in Supplementary Table 2. Then, the MethylKIT package in R 3.5.1 was used to analyze the methylation data and calculate the average methylation level at all CpG sites. The overall methylation level of each gene was calculated by the average value of the methylation levels of all the CpG sites located in the promoter region.

### Quantitative Real-Time Polymerase Chain Reaction

The primers used for PCR amplification are listed in Supplementary Table 1. Total RNA was extracted using TRIzol reagent (TAKARA). The cDNA reverse transcription kit (TOYOBO) was used to reverse-transcribe RNA, and the SYBR Green PCR Kit (Applied Biosystems) was utilized to amplify the resulting cDNA. The samples were detected by the Applied Biosystems StepOne Real-Time PCR System. GAPDH mRNA levels were quantified as a reference control, and all samples were run in triplicate. Relative mRNA expression levels of the MDGs were determined using the 2^–ΔΔ*Ct*^ method, and the FC was normalized against the mRNA levels of the GAPDH gene.

## Results

### Identification of DEGs in GBM

A total of 13,625 DEGs, including 6,565 upregulated and 7,060 downregulated genes, were identified between GBM and normal samples in the TCGA GBM dataset, which were selected for further analysis.

### Identification of MDGs and Enrichment Analyses

**FIGURE 1:**
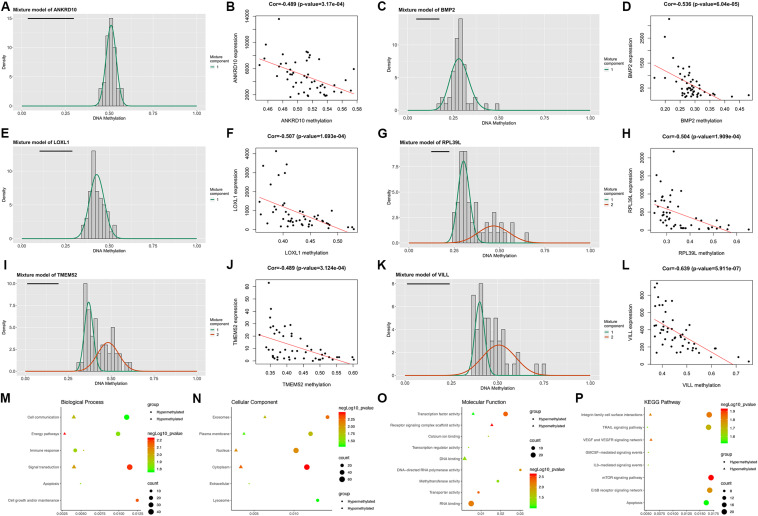
Overview of the DNA methylation-driven genes (MDGs) identified by MethylMix analysis. **(A,C,E,G,I,K)** The β mixture models of the representative six MDGs. The distribution of methylation status among GBM samples is shown by the histogram. The distribution of methylation status among normal samples is shown by the black line. **(B,D,F,H,J,L)** Correlation plots of the methylation and expression levels of the representative six MDGs. Biological process **(M)**, cellular component **(N)**, and molecular function **(O)** terms and KEGG pathways **(P)** enriched in the 199 MDGs.

Following the MethylMix algorithm and independent sample *t*-test analysis, we identified a total of 199 aberrantly methylated and differentially expressed MDGs (Supplementary Table 3). Most of these MDGs (146/199, 73.4%) were highly methylated; only 53 (26.7%) genes were lowly methylated (Figure 1). The β mixture models and correlation plots of the representative MDGs are displayed in Figure 1.

Then, enrichment analyses were performed to explore the molecular mechanisms of MDGs in the development and progression of GBM. In the BP category, the MDGs were significantly enriched in signal transduction, cell communication and energy pathways (Figure 1M). In the CC category, the MDGs were significantly enriched in the cytoplasm, nucleus, plasma membrane and exosomes (Figure 1N). In the MF category, the high-methylated MDGs were significantly enriched in RNA binding and methyltransferase activity, whereas the low-methylated genes were enriched in DNA binding and transcription factor activity (Figure 1O). In addition, KEGG pathway analysis revealed that the high-methylated MDGs were mainly enriched in integrin family cell surface interactions, mTOR signaling pathway, and apoptosis, whereas the low-methylated genes were enriched in integrin family cell surface interactions and the VEGF and VEGFR signaling network (Figure 1P).

### MDG-Based Molecular Classification of GBM Patients and Associations With Prognosis and Clinical Patterns

**FIGURE 2:**
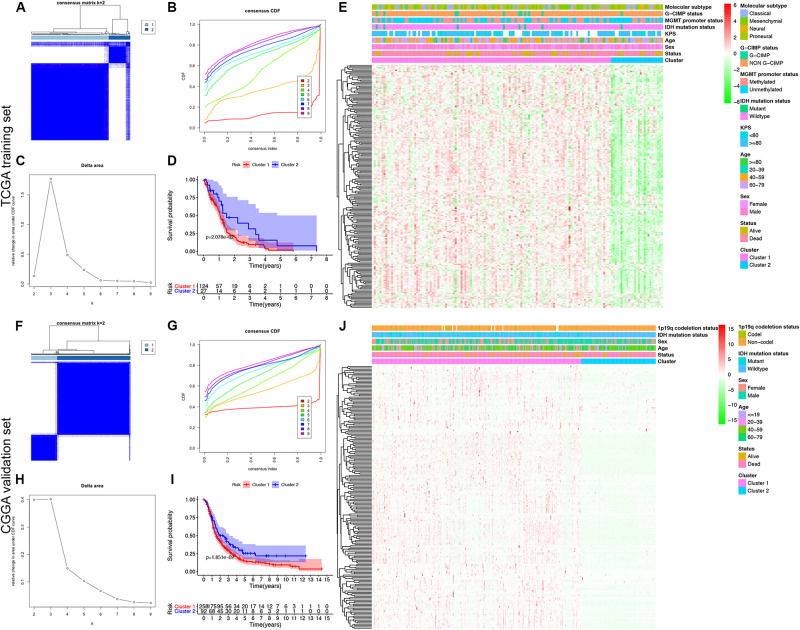
Identification and validation of an MDG-based molecular classification of GBM patients using the unsupervised consensus clustering algorithm. Consensus clustering matrix for *k* = 2, which was the optimal cluster number in both the TCGA training cohort **(A)** and CGGA validation cohort **(F)**. Cumulative distribution function (CDF) curves of the consensus score (*k* = 2–9) in the TCGA **(B)** and CGGA cohorts **(G)**. The relative change in the area under the CDF curve (*k* = 2–9) in the TCGA **(C)** and CGGA cohorts **(H)**. Kaplan-Meier survival analyses of the patients in the Cluster 1 and Cluster 2 subgroups in the TCGA **(D)** and CGGA cohorts **(I)**, which indicated that the patients in Cluster 1 had poorer OS than those in Cluster 2. The heatmap and clinicopathological features of the two clusters based on the expression patterns of the MDGs in the TCGA **(E)** and CGGA cohorts **(J)**.

To explore a novel molecular classification of GBM based on the expression patterns of the MDGs, unsupervised consensus clustering was performed on the 151 TCGA GBM patients. According to the relative change in the area under the CDF curve and consensus heatmap, the optimal number of clusters was determined as two (*k* value = 2), and no appreciable increase was observed in the area under the CDF curve (Figures 2A–C). Then, all 151 patients were divided into two subgroups, including 124 (82.1%) patients in Cluster 1 and 27 (17.9%) in Cluster 2. K-M survival analysis demonstrated that patients in Cluster 1 showed significantly worse OS than those in Cluster 2 (log-rank *P* = 2.078 × 10^–2^; Figure 2D). Then, the same method was applied to validate the molecular classification in the CGGA GBM patients. As shown in Figures 2F–H, the optimal number of clusters was again determined as two (*k* value = 2), and the 350 GBM patients were divided into Cluster 1 (258 patients, 73.7%) and Cluster 2 (92 patients, 26.3%). The patients in Cluster 1 again showed significantly worse OS than those in Cluster 2 (log-rank *P* = 1.851 × 10^–2^; Figure 2I). Subgroup analysis demonstrated that when stratifying patients by age, sex, IDH mutation status, and other clinical variables, Cluster 1 patients had worse survival than Cluster 2 (HR > 1 and *P* < 0.05; Supplementary Figure 2). Then, the cluster quality measure was applied to verify the similarities between the different subgroups. The IGP score of TCGA Cluster 1 was 0.811 and that of TCGA Cluster 2 was 0.198 (*P* < 0.001), whereas the IGP score of CGGA Cluster 1 was 0.832 and that of CGGA Cluster 2 was 0.144 (*P* < 0.001). There was no significant difference between TCGA Cluster 1 and CGGA Cluster 1 (*P* = 0.831) or between TCGA Cluster 2 and CGGA Cluster 2 (*P* = 0.998).

Finally, we also analyzed the expression patterns of the MDGs and compared the clinicopathological variables between two clusters of GBM patients. The expression patterns of the GBM-specific MDGs were visualized in heat maps, which are shown in Figure 2E (TCGA) and Figure 2J (CGGA). Generally, the expression levels of most MDGs in Cluster 1 were significantly upregulated compared with those in Cluster 2 in both the TCGA and CGGA GBM cohorts, which indicated that an increase in the expression levels of the MDGs may be correlated with poor prognosis. The comparisons of the clinicopathological variables between two clusters of GBM patients are shown in Supplementary Figure 1. Compared with Cluster 2, the patients in Cluster 1 were significantly older in both the TCGA (*P* = 0.013) and CGGA cohort (*P* = 0.009). Additionally, more 1p/19q codeletions were observed in Cluster 1 patients in the CGGA cohort (*P* = 0.018). However, no significant difference in the other clinicopathological factors was observed between the two clusters (all *P* > 0.05). Overall, the patients in the Cluster 1 subgroup, with high MDG expression patterns and older age, commonly exhibited a poor prognosis. These findings demonstrated that our novel MDG-based molecular classification of GBM was robust and reliable in different populations, and different survival outcomes and clinicopathological parameters can be clearly discriminated.

### Predictions of Immunotherapy Response of the GBM Patients

**FIGURE 3:**
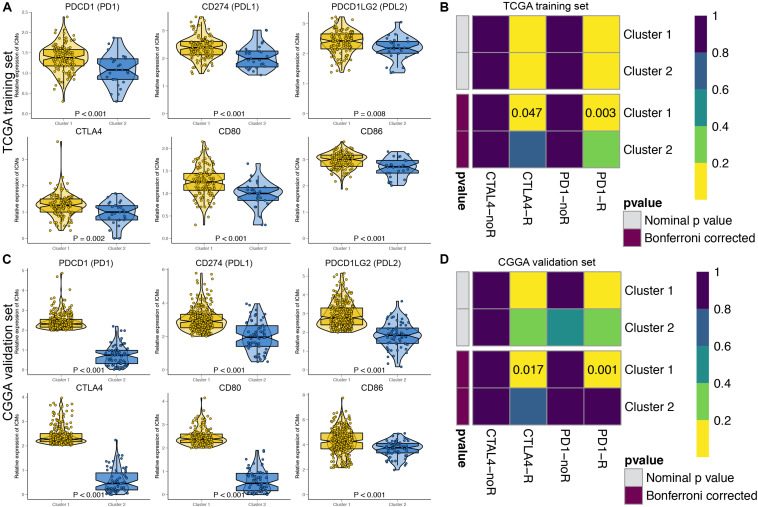
The expressions of immune checkpoint molecules and predictions of immunotherapy response of the GBM patients in two MDG-based molecular subgroups. The expressions of immune checkpoint molecules were significantly higher in Cluster 1 patients than in Cluster 2 patients in both the TCGA **(A)** and CGGA **(C)** cohort. Subclass mapping analysis of the TCGA **(B)** and CGGA **(D)** GBM patients for predicting the likelihood of clinical response to anti-PD1 and anti-CTLA4 therapy in two clusters. R, immunotherapy respondent.

The immune checkpoint molecules, including PDCD1 (PD1), CD274 (PDL1), PDCD1LG2 (PDL2), CTLA4, CD80, and CD86, were all significantly highly expressed in Cluster 1 patients in both the TCGA (Figure 3A) and CGGA cohorts (Figure 3C). The TIDE algorithm was applied to predict the likelihood of immunotherapy response of each MDG-based molecular cluster of GBM patients. In the TCGA training cohort, Cluster 1 (33.9%, 42/124) patients were more likely to respond to immunotherapy than Cluster 2 (18.5%, 5/27) patients (*P* < 0.001). Similarly, in the CGGA validation cohort, Cluster 1 (31.7%, 91/287) patients were also more sensitive to immunotherapy than Cluster 2 (15.9%, 10/63) patients (*P* < 0.001). Then, SubMap analysis was further used to predict the likelihood of a clinical response to anti-PD1 and anti-CTLA4 therapy in the two clusters (Figure 3). SubMap analysis demonstrated that compared with Cluster 2 GBM patients, Cluster 1 patients in both the TCGA and CGGA cohorts were more sensitive to CTLA4 and PD1 inhibitors (Figures 3B,D).

### Construction and Validation of the MDG-Based Prognostic Risk Score Model (MDG Signature)

By performing the univariate Cox regression analysis on the 199 candidate genes in the TCGA GBM cohort, we identified 29 prognosis-related MDGs. Then, 10 out of 29 MDGs were further screened by the LASSO regression analysis (Supplementary Figure 3). Subsequently, six MDGs were finally selected by the multivariate Cox analysis as the significant prognostic genes, including (ANKRD10, HR = 0.53), (BMP2, HR = 0.52), (LOXL1, HR = 1.81), (RPL39L, HR = 1.72), (TMEM52, HR = 1.34), and (VILL, HR = 2.13). The expression levels of the 6 MDGs between GBM and normal tissues were further validated in the GEPIA database (163 tumor samples and 207 normal cerebral samples), which revealed that all the 6 MDGs were expressed at low levels in the GBM samples (Supplementary Figure 4). Notably, all the 6 MDGs were significantly highly methylated in the GBM samples compared with the normal cerebral samples (Supplementary Figure 4).

**FIGURE 4:**
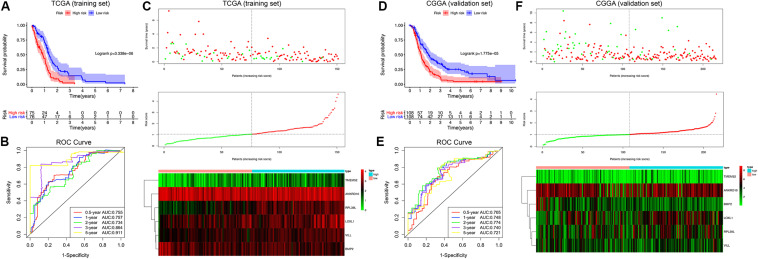
Survival analysis, prognostic performance and risk score analysis of the MDG-based risk score model in GBM. K-M survival analysis was performed to estimate the prognosis of patients with high risk scores and low risk scores in the TCGA training cohort **(A)** and CGGA validation cohort **(D)**. The high-risk groups had significantly poorer OS than the low-risk groups. The prognostic performances of the MDG signature demonstrated by the time-dependent ROC curve for predicting the 0. 5-, 1-, 2-, 3-, and 5-year OS rates in the TCGA training cohort **(B)** and CGGA validation cohort **(E)**. Risk score analysis of the MDG signature in the TCGA training cohort **(C)** and CGGA validation cohort **(F)**. Upper panel: Patient survival status and time distributed by risk score. Middle panel: Risk score curves of the MDG signature. Bottom panel: Heat maps of the expressions of the 6 MDGs in the GBM samples. The colors from green to red indicate the expression level from low to high.

Afterward, the MDG-based prognostic risk score model was established with the following formula: risk score = Exp_*ANKRD*__10_ × (−0.628) + Exp_*BMP*__2_ × (−0.644) + Exp_*LOXL*__1_ × 0.595 + Exp_*RPL*__39__*L*_ × 0.544 + Exp_*TMEM*__52_ × 0.291 + Exp_*VILL*_ × 0.754 (Supplementary Figure 3C). Then, we calculated the risk score for each patient in the TCGA training cohort. All patients were divided into high-risk (high risk score) and low-risk (low risk score) groups using the median value of the risk score as the cutoff (Figure 4C). K-M survival analysis indicated that patients with high risk scores demonstrated significantly poorer OS than patients with low risk scores (logrank *P* = 3.338 × 10^–6^; Figure 4A). The C-index of the MDG-based prognostic model for OS prediction was 0.802 (95% CI, 0.763 to 0.841; *P* = 4.33 × 10^–21^). Additionally, by performing the time-dependent ROC analysis, the MDG signature also showed favorable values in predicting 0. 5-, 1-, 3- and 5-year OS rates, with respective AUC values of 0.755, 0.757, 0.864 and 0.911 in the TCGA GBM training set (Figure 4B).

Finally, to evaluate whether the MDG-based prognostic model had similar predictive performances in different populations, we applied it to predict OS in an independent external validation cohort in a similar manner. According to the risk score model, the 216 GBM patients from CGGA dataset were divided into high-risk and low-risk groups (Figure 4F). The OS of patients with high risk scores was significantly poorer than that of those with low risk scores (logrank *P* = 1.775 × 10^–5^; Figure 4D). The MDG signature also showed a favorable predictive ability of the 0. 5-, 1-, 3- and 5-year OS rates, with AUC values of 0.705, 0.748, 0.740 and 0.721, respectively, in the CGGA validation set (Figure 4E). These results demonstrated that the MDG signature may serve as a robust and reliable prognostic predictor for GBM patients from different populations.

### Determination of the MDG Signature as an Independent Prognostic Factor

Table 1 shows the demographics and clinicopathological characteristics of GBM patients with high and low risk scores in the TCGA training cohort and CGGA validation cohort based on the MDG signature. Univariate and multivariate Cox regression analyses were performed to evaluate the prognostic significance of the MDG signature independent of other clinicopathological parameters (Table 2). In the TCGA training cohort, univariate Cox regression analysis demonstrated that age (*P* = 1.98 × 10^–4^), new event (*P* = 2.81 × 10^–3^), pharmacotherapy (*P* = 6.97 × 10^–5^), radiotherapy (*P* = 1.04 × 10^–3^), IDH mutation status (*P* = 8.91 × 10^–3^), MGMT promoter methylation status (*P* = 6.84 × 10^–3^), ATRX mutation status (*P* = 4.28 × 10^–2^), and MDG signature (*P* = 6.27 × 10^–6^) significantly correlated with prognosis (Table 2). Then, the significant survival-associated parameters were enrolled in the multivariate analysis, which indicated that age (*P* = 1.56 × 10^–2^), new event (*P* = 4.31 × 10^–2^), pharmacotherapy (*P* = 2.04 × 10^–2^), radiotherapy (*P* = 4.78 × 10^–3^), IDH mutation status (*P* = 3.42 × 10^–2^), MGMT promoter methylation status (*P* = 1.39 × 10^–2^), and MDG signature (*P* = 1.25 × 10^–4^) were significantly associated with OS (Table 2). Additionally, following the univariate and multivariate Cox regression analyses, MDG signature was also proven to be an independent prognostic predictor in the CGGA validation set (Table 2). Therefore, the MDG-based prediction model constructed by the TCGA training set may serve as an independent prognostic factor for GBM patients in different populations.

**TABLE 1:** Demographics and clinicopathological characteristics of GBM patients in the TCGA training cohort and CGGA validation cohort based on the DNA methylation-driven gene (MDG) signature.

**Variables**	**TCGA cohort (training set)**			**CGGA cohort (validation set)**		
	**Total (*n* = 151)**	**Low risk (*n* = 76)**	**High risk (*n* = 75)**	**Total (*n* = 216)**	**Low risk (*n* = 108)**	**High risk (*n* = 108)**
Age (years)	59.6 ± 13.7	57.7 ± 12.9	61.5 ± 14.3	48.8 ± 13.9	46.3 ± 14.1	50.2 ± 13.5
**Sex**						
Female	53	23	30	86	50	36
Male	98	53	45	130	58	72
**New event**						
No	64	28	36	85	40	49
Yes	87	48	39	131	68	59
**KPS**						
<80	32	15	17	NA		
≥80	81	44	37	NA		
NA	38	17	21	NA		
**Pharmacotherapy**						
TMZ	64	31	33	40 (No)	18	22
TMZ + BEV	26	12	14	168 (Yes)	85	83
Others (No TMZ)	19	10	9	–	–	–
No or NA	42	23	19	8 (NA)	5	3
**Radiotherapy**						
No	22	7	15	26	14	12
Yes	122	66	56	183	90	93
NA	7	3	4	7	4	3
**Surgery**						
Biopsy only	16	9	7	NA		
Tumor resection	135	67	68	NA		
**IDH status**						
Wildtype	147	68	75	182	78	104
Mutant	8	8	0	34	30	4
**MGMT promoter status**						
Methylated	66	26	40	105	37	55
Unmethylated	85	50	35	111	71	53
**TERT status**						
Wildtype	146	74	72	NA		
Mutant	5	2	3	NA		
**BRAF status**						
Wildtype	146	75	71	NA		
Mutant	5	1	4	NA		
**ATRX status**						
Wildtype	140	67	73	NA		
Mutant	11	9	2	NA		
**EGFR status**						
Wildtype	97	48	49	NA		
Mutant	54	28	26	NA		
**1p/19q status**						
Non-codeletion	NA			185	104	81
Codeletion	NA			5	4	1
NA	NA			26	1	25

*GBM, glioblastoma; MDG, DNA methylation-driven gene; NA, not available; KPS, Karnofsky performance score; TMZ, temozolomide; BEV, bevacizumab; PCV, procarbazine lomustine vinCRISTine. “New event” included progression and recurrence. “Others (No TMZ)” in pharmacotherapy included PCV, PCV+BEV, and other drugs, including avastin, carmustine, and irinotecan.*

**TABLE 2:** Univariate and multivariate cox proportional hazards analysis of clinicopathological parameters and MDG signature of GBM patients in the TCGA training cohort and CGGA validation cohort.

**Variables**	**TCGA cohort (training set)**				**CGGA cohort (validation set)**			
	**Univariate analysis**		**Multivariate analysis**		**Univariate analysis**		**Multivariate analysis**	
	**HR (95% CI)**	***P*-value**	**HR (95% CI)**	***P*-value**	**HR (95% CI)**	***P*-value**	**HR (95% CI)**	***P*-value**
Age	1.03 (1.01–1.04)	**1.98e–04**	1.05 (1.02–1.08)	**1.56e–02**	1.21 (1.19–1.26)	**9.53e–03**	1.34 (1.30–1.38)	**2.33e–02**
Sex	0.92 (0.63–1.34)	0.65	–	–	1.29 (0.95–1.77)	0.11	–	–
New event	0.56 (0.39–0.82)	**2.81e–03**	0.63 (0.41–0.98)	**4.31e–02**	0.79 (0.66–0.92)	**1.88e–05**	0.85 (0.77–0.93)	**3.11e–02**
KPS	0.93 (0.69–1.23)	0.59	–	–	NA		NA	
Pharmacotherapy	1.27 (1.13–1.42)	**6.97e–05**	1.16 (1.02–1.32)	**2.04e–02**	1.55 (1.16–1.94)	**1.98e–03**	1.49 (1.19–1.79)	**3.51e–04**
Radiotherapy	0.43 (0.26–0.71)	**1.04e–03**	0.48 (0.29–0.79)	**4.78e–03**	0.73 (0.53–0.93)	**1.97e–03**	0.77 (0.74–0.81)	**1.88e–02**
Surgery	0.93 (0.52–1.67)	0.82	–	–	NA		NA	
IDH status	0.26 (0.09–0.71)	**8.91e–03**	0.18 (0.04–0.88)	**3.42e–02**	0.77 (0.63–0.95)	**1.23e–02**	0.89 (0.86–0.92)	**2.88e–02**
MGMT promoter status	1.43 (1.13–1.73)	**6.84e–03**	1.37 (1.07–1.67)	**1.39e–02**	2.57 (2.04–4.54)	**2.48e–05**	1.49 (1.31–1.82)	**4.21e–02**
TERT status	0.91 (0.29–2.86)	0.87	–	–	NA		NA	
BRAF status	1.97 (0.72–5.41)	0.19	–	–	NA		NA	
ATRX status	0.43 (0.19–0.97)	**4.28e–02**	2.38 (0.63–9.01)	0.20	NA		NA	
EGFR status	1.27 (0.87–1.86)	0.21	–	–	NA		NA	
1p/19q status	NA		NA		0.77 (0.24–2.43)	0.65	–	–
MDG signature	2.41 (1.64–3.53)	**6.27e–06**	1.92 (1.29–2.85)	**1.25e–04**	1.93 (1.42–2.63)	**2.38e–05**	1.94 (1.39–2.70)	**9.05e–05**

*MDG, DNA methylation-driven gene; GBM, glioblastoma; HR, hazard ratio; CI, confidence interval; KPS, Karnofsky performance score. All statistical tests were two-sided. Bold type means *P* < 0.05.*

### Construction and Validation of the Nomogram

**FIGURE 5:**
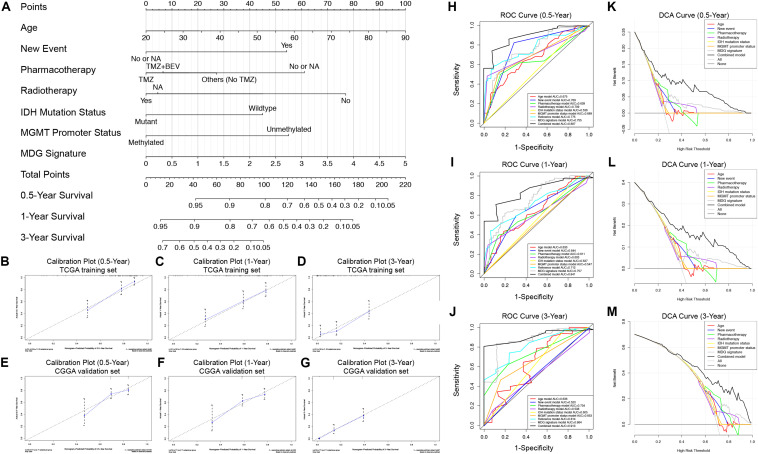
Prognostic nomogram used to predict the 0. 5-, 1–, and 3–year survival probability of GBM patients. **(A)** Nomogram model used to predict the survival of GBM patients based on the TCGA training cohort. Calibration plots of the nomogram for predicting survival at 0.5, 1, and 3 years in the TCGA training cohort **(B–D)** and CGGA validation cohort **(E–G)**. The actual survival is plotted on the *y*-axis; the nomogram-predicted probability is plotted on the *x*-axis. **(H–J)** The prognostic performances of the nomogram demonstrated by the ROC curve for predicting the 0. 5-, 1–, and 3–year OS rate compared with other clinicopathological factor-based prognostic models. **(K–M)** The clinical benefit and the scope of applications of the nomogram evaluated by the DCA curves at 0.5, 1, and 3 years. The net benefit is plotted on the *y*-axis, and the threshold probabilities of patients having 1–, 3– and 5–year survival is plotted on the *x*-axis.

To generate a clinically applicable model for individual OS prediction, we successfully constructed a prognostic nomogram to predict the probability of 0. 5-, 1−, and 3−year survival of GBM patients. Firstly, 6 independent prognostic factors, including age, new event, pharmacotherapy, radiotherapy, IDH mutation status, and MGMT promoter methylation status were used to construct a prediction model as the reference model. Then, 7 independent prognostic factors, including 6 clinical variables and MDG signature, were enrolled into the final prediction model (Figure 5A). The Schoenfeld Residuals Test demonstrated *P* > 0.05 for all the clinical variables and combined model, and thereby PH assumption can be satisfied and Cox regression analysis was reliable as a result (Supplementary Figure 5). The C-index of the nomogram was 0.855 (95% CI, 0.816 to 0.894; *P* = 8.71 × 10^–30^). The calibration plots showed excellent agreement between the predicted 0. 5-, 1- and 3-year survival rates and actual observations in the TCGA cohort (Figures 5B–D). ROC curve analysis indicated favorable predictive abilities of 0. 5-, 1- and 3-year OS rates, with AUC values of 0.887, 0.841 and 0.913, respectively (Figures 5H–J). In addition, DCA curve analysis was used to determine the clinical usefulness of the prognostic nomogram, which showed the best net benefit at 0.5, 1, and 3 years compared with other prognostic models (Figures 5K–M). ROC and DCA analyses demonstrated that the discrimination performance of the nomogram was significantly better than that of the other prognostic models constructed by a single factor and the reference model with only clinical variables (Figures 5H–J). All the above-mentioned findings suggested the appreciable reliability of the prognostic nomogram constructed by the TCGA training set. In addition, in the CGGA external validation cohort, the C-index of the nomogram for predicting the survival of 216 GBM patients was 0.776 (95% CI, 0.737 to 0.815; *P* = 3.31 × 10^–15^). The calibration plots also indicated excellent agreement between survival prediction and actual observation in the probabilities of 0. 5-, 1- and 3-year OS in the CGGA cohort (Figures 5E–G). The nomogram achieved an AUC of 0.791, 0.752, and 0.833 for 0. 5-, 1-, and 3-year OS, respectively, in the CGGA validation cohort (Supplementary Figure 6).

### Integrated Survival Analyses Based on the Expression and Methylation of the Six MDGs

**FIGURE 6:**
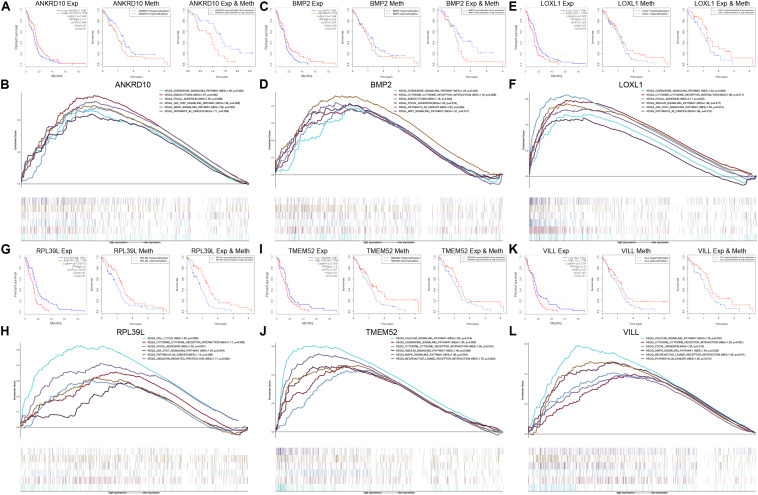
GSEA and integrated survival analyses based on the expression and methylation of the 6 MDGs in GBM patients. K-M survival analyses of ANKRD10 **(A)**, BMP2 **(C)**, LOXL1 **(E)**, RPL39L **(G)**, TMEM52 **(I)**, and VILL **(K)**. Left panel: Survival analyses based on the expression levels of the 6 MDGs. Middle panel: Survival analyses based on the methylation levels of the 6 MDGs. Right panel: Integrated survival analyses based on gene expression and methylation of the 6 MDGs to assess the survival differences between high methylation, low expression patients and low methylation, and high expression patients in the TCGA GBM cohort. GSEA analyses of the 6 MDGs in the TCGA GBM cohort. Enriched KEGG pathways of the 6 MDGs are listed in the upper right. Exp, gene expression; Meth, DNA methylation; NES, normalized enrichment score; p, nominal *p*-value.

To further explore the prognostic values of the 6 MDGs enrolled in the prediction model, K-M survival analyses were performed to assess the associations between the gene expression/DNA methylation levels and OS. The ANKRD10 and BMP2 high expression group and LOXL1, RPL39L, TMEM52 and VILL low expression group had a better prognosis (Figure 6). In addition, the ANKRD10 and BMP2 low-methylation group and LOXL1, RPL39L, TMEM52 and VILL high-rmethylation group had a better prognosis (Figure 6). Finally, the integrated survival analyses were performed, and the results demonstrated that the low methylation/high expression survival rates of ANKRD10 and BMP2 were significantly higher, whereas the high methylation/low expression survival rates of LOXL1, RPL39L, TMEM52 and VILL were significantly higher (Figure 6; Supplementary Table 4). These findings suggested that the 6 MDGs all exhibited excellent prognostic values in discriminating GBM patients based on the gene expression and DNA methylation.

### GSEA of the Six MDGs

GSEA revealed that high expressions of the 6 MDGs that were significantly enriched in the KEGG pathways were related to the development, progression and metastasis of tumors, including pathways in cancer, focal adhesion, cytokine-cytokine receptor interaction, the chemokine signaling pathway, the MAPK signaling pathway, and the JAK-STAT signaling pathway (Figure 6). These findings strongly indicated the potential role of the MDGs in the tumorigenesis and progression of GBM, which may provide new evidence for cancer-targeted treatments for GBM patients.

### Validations of the Associations Between Promoter Methylations and Expressions of the Six MDGs

**FIGURE 7:**
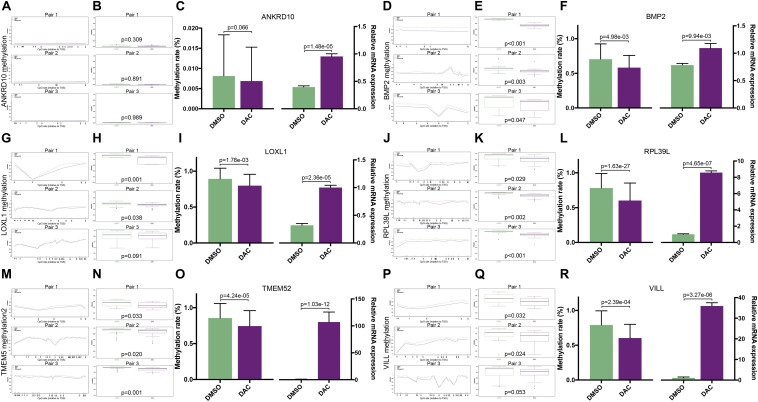
The associations between promoter methylations and expressions of the six MDGs quantified by BSAS and qPCR. The methylation levels of the relative CpG sites in 3 primer regions (Pair 1, 2, and 3) of each MDG in the DMSO and DAC groups, including ANKRD10 **(A)**, BMP2 **(D)**, LOXL1 **(G)**, RPL39L **(J)**, TMEM52 **(M)**, and VILL **(P)**. The green dots represent the DMSO control group, and the red dots represent the DAC treatment group. Asterisks (*) indicate *P* < 0.05. The combined methylation levels of all CpG sites located in one primer region of the MDGs in the DMSO and DAC groups, including ANKRD10 **(B)**, BMP2 **(E)**, LOXL1 **(H)**, RPL39L **(K)**, TMEM52 **(N)**, and VILL **(Q)**. The green boxes represent the DMSO control group, and the red boxes represent the DAC treatment group. The combined methylation levels of all CpG sites in the 3 primer regions (Left panel) and the relative expression levels (Right panel) of the six MDGs in the DMSO and DAC groups, including ANKRD10 **(C)**, BMP2 **(F)**, LOXL1 **(I)**, RPL39L **(L)**, TMEM52 **(O)**, and VILL **(R)**.

To validate whether the expression of the six MDGs was indeed regulated by the promoter region methylation, BSAS and qPCR were, respectively, performed to detect their methylation and expression levels. As shown in Figure 1, there were strong negative correlations between methylation and mRNA expression levels of the six MDGs. Thus, to assess whether the changes in promoter methylation status were associated with gene expression, we applied DAC, a demethylation agent, to treat GBM cells *in vitro*. As shown in Figure 7, compared with the DMSO control group, the methylation levels of the CpG sites in each primer region of each MDG were mostly decreased in the DAC treatment group, except for ANKRD10, which demonstrated rather low methylation status in both the DAC and DMSO groups. Generally, after combing all the CpG sites located in the promoter region, the overall methylation levels of the promoter were all significantly decreased after DAC treatment (all *P* < 0.001), except for ANKRD10 (*P* = 0.066). In contrast, remarkable restorations of gene expression were observed in all six MDGs (all *P* < 0.001).

## Discussion

Epigenetic alterations have been widely reported to be crucial components of the oncogenesis and progression of multiple cancers (Kulis and Esteller, 2010; Wilting and Dannenberg, 2012). Aberrant DNA methylation could cause cell differentiation disorders and transcriptional disorders, including decreased expression of genes via high methylation and increased expression via low methylation, and plays a key role in the tumorigenesis, recurrence, and drug resistance of GBM (Baylin and Jones, 2011; Klughammer et al., 2018; Aoki and Natsume, 2019). De Souza et al. (2018) reported a pronounced loss of DNA methylation during the progression and recurrence of glioma. Klughammer et al. (2018) reported subtle differences between primary and recurrent GBM and links between DNA methylation and the tumor microenvironment. In addition, altered DNA methylation events could also act as a prognostic predictor and therapeutic target for GBM. Hegi et al. (2005) reported that GBM patients with MGMT promoter methylation exhibited better OS and increased time to progression of the disease after chemotherapy and/or radiotherapy. DNA methylation targeted therapies were also reported to be applied in some clinical and preclinical studies. A phase I study for 5-azacytidine, a DNA methylation inhibitor, is underway for patients with GBM (Aoki and Natsume, 2019). Thus, DNA methylation and MDGs can be widely used for early diagnosis, risk stratification, prognosis prediction, and therapeutic targets for GBM.

In the present study, a multiomic analysis based on transcriptomic and DNA methylation profiles was performed to develop global DNA methylation patterns, which has never been realized in GBM before in the literature. First, we identified 199 aberrantly methylated and differentially expressed MDGs by using the MethylMix algorithm. Pathway enrichment analysis indicated that MDGs were mainly involved in cancer-related pathways, which indicated that abnormal DNA methylation and aberrant MDGs could play a vital role in the tumorigenesis and progression of GBM. Then, we developed a novel molecular classification of GBM patients based on the expression patterns of MDGs, which was then validated by the CGGA cohort. The patients in the Cluster 1 subgroup, with high expression patterns of the MDGs and older age, commonly exhibited poor prognosis. Our findings demonstrated that GBM patients from different populations can be reliably classified into two subgroups based on the 199 MDGs, and different survival outcomes and clinicopathological patterns can be clearly discriminated by our novel molecular classifications. TIDE and SubMap analysis demonstrated that Cluster 1 patients were more sensitive to anti-CTLA4 and anti-PD1 therapies. Thus, for those GBM patients in Cluster 1 with a poor prognosis, immunotherapy may be applied more aggressively and earlier to improve the prognosis of those patients more effectively. Compared with the traditional molecular subtyping of GBM, dividing patients into 4 subgroups based on the whole transcriptomic data, the novel MDG-based subtyping was based on only 199 genes, and can well distinguish 2 groups with distinct expression patterns, which is more convenient for its clinical application. In addition, the MDG-based classification was strongly correlated to OS and immunotherapy response, which were unique advantages that traditional molecular classification did not have. Then, univariate, LASSO and multivariate Cox regression analysis were, respectively, applied to identify the prognosis-associated MDGs. The integrated survival analyses demonstrated that the 6 MDGs all exhibited excellent prognostic values in discriminating low methylation/high expression groups and high methylation/low expression groups in GBM. GSEA also revealed that high expressions of the 6 MDGs were significantly enriched in the KEGG pathways related to the development, progression and metastasis of tumors. All these findings strongly indicated the promising values of the MDGs in survival prediction and cancer-targeted treatments for GBM.

In addition, *in vitro* BSAS and qPCR analysis demonstrated that the expression of the six MDGs was negatively regulated by promoter region methylation in GBM cell lines. Our findings validated that the six genes were driven by DNA methylation and strongly suggested that these MDGs deserve further investigation to clarify their potential roles in the development and progression of GBM. ANKRD10 is a protein coding gene, which has not been well studied in the literature. Ji et al. (2018) reported that morin treatment resulted in anti-tumor activity *in vitro* by downregulating the expression of ANKRD10 in tongue squamous cell carcinoma cells. In our study, ANKRD10 was highly expressed in GBM and acted as a favorable prognostic factor. However, no other studies explored the roles of ANKRD10 and its methylation in the development of glioma. BMP2, which encodes a secreted ligand of the transforming growth factor-β (TGF-β) superfamily of proteins, is known to promote differentiation and growth inhibition in GBM cells (Piccirillo et al., 2006; Pistollato et al., 2009). Persano et al. (2012) reported that BMP2 can increase GBM responsiveness to temozolomide by downregulating the HIF-1α/MGMT axis and can serve as a favorable predictor of survival, which is consistent with our study. LOXL1, which encodes a matrix cross-linking enzyme, has been identified as closely associated with the tumorigenesis of multiple cancers, including non-small cell lung cancer, breast cancer, and urological cancer (Jeong et al., 2018; Zeltz et al., 2019). Wu et al. (2007) demonstrated that LOXL1 is epigenetically silenced by promoter hypermethylation and can inhibit the Ras/ERK signaling pathway in bladder cancers. In addition, LOXL1 antisense RNA 1 (LOXL1-AS1) was reported to clinically serve as a poor prognostic indicator and able to contribute to aggressive behaviors related to the mesenchymal subtype of GBM via the NF-κB signaling pathway (Wang et al., 2018). RPL39L encodes a protein sharing high sequence similarity with ribosomal protein L39. It was reported to show highly specific tissue expression patterns in multiple tumors, such as hepatocellular carcinoma (Wong et al., 2014). Devaney et al. (2013) found that RPL39L methylation was associated with inactivation of gene expression in prostate cancer cell lines. TMEM52 encodes a transmembrane protein and is largely modified by DNA methylation (Almeida et al., 2018). Both RPL39L and TMEM52 served as unfavorable prognostic factors in our study. However, no previous studies have investigated the roles of RPL39L and TMEM52 in GBM. VILL encodes proteins belonging to the villin/gelsolin family. Grzendowski et al. (2010) reported that aberrant methylation of the 5′-CpG islands contributed to epigenetic down-regulation of VILL in 1p/19q-deleted gliomas. Our study demonstrated that high expression of VILL was associated with poor prognosis of GBM patients, which has never been reported in previous studies. Considering that methylation is potentially reversible, detection of those aberrant methylated MDGs, including ANKRD10, BMP2, LOXL1, RPL39L, TMEM52, and VILL, may become potential molecular therapeutic targets for the treatment of GBM. Therefore, corresponding drugs can be developed to prevent or even reverse the tumorigenesis and progression of tumor cells by correcting abnormal DNA methylation.

Then, a novel prognostic prediction model based on the six MDGs was successfully constructed and validated in separate patient populations. The MDG signature was identified to be an independent prognostic factor compared with other clinicopathological factors and demonstrated favorable predictive value in discriminating high- and low-risk GBM patients with significantly different survival outcomes. Thus, the novel prognostic signature can be used for individualized survival prediction and development of treatment strategies for GBM patients. More aggressive treatments and closer follow-ups should be applied in those patients with high risk scores. Additionally, in this study, we found that most of traditional molecular biomarkers were not independent predictors for prognosis. Only IDH mutations and MGMT promoter methylation status were determined as independent predictors for OS, and they only showed poor predictive value with AUC < 0.7, far less than the performances of the MDG signature. Therefore, compared with those traditional molecular biomarkers, the MDG-based prognostic models were much more robust and reliable in predicting survival of GBM patients.

Due to the intuitive visual presentation of the nomogram model, it has been widely utilized to construct prediction models for clinical practice (Harrell et al., 1996; Balachandran et al., 2015). To the best of our knowledge, this is the first prognostic nomogram with a global DNA methylation signature that was constructed by large-scale GBM databases with long-term follow-up. In this study, we constructed a nomogram with age, new event, pharmacotherapy, radiotherapy, IDH mutation status, MGMT promoter methylation status and MDG signature. The calibration plots and ROC curves demonstrated the excellent and reliable predictive performance of the nomogram in both the TCGA training cohort and CGGA validation cohort. In addition, following the evaluation of clinical usefulness by DCA curves, our visualized scoring system showed appreciable reliability in assisting physicians in developing individualized prognostic prediction and treatment strategies, which could facilitate better treatment decision-making and follow−up scheduling.

In conclusion, by performing a combined multiomic analysis based on transcriptomic and DNA methylation profiles, we first identified the aberrantly methylated and differentially expressed DNA MDGs by using the MethylMix algorithm. Then, we developed and validated a novel MDG-based molecular classification of GBM, which was associated with prognosis and immunotherapy response. A reliable MDG-based risk score model was further identified for risk stratification, survival prediction, and therapeutic targets for GBM. Furthermore, a novel promising prognostic nomogram with MDG signature, age, new event, pharmacotherapy, radiotherapy, IDH mutation status, and MGMT promoter methylation status was successfully developed for individualized prognosis prediction to facilitate the development of better treatment strategies and follow−up scheduling. *In vitro* BSAS and qPCR analysis validated that demethylation in general including the promoter regions of MDGs would contribute to higher expression of the target genes. Multicenter, large-scale clinical trials and prospective studies are needed to further validate the prognostic prediction model in this study.

## Data Availability Statement

All datasets presented in this study are included in the article/Supplementary Material.

## Author Contributions

LG and XG performed the data curation and analysis. KD and WL analyzed and interpreted the results. ZW and BX drafted and reviewed the manuscript. BX designed the study and critically revised it for important intellectual content. All authors reviewed the manuscript.

## Conflict of Interest

The authors declare that the research was conducted in the absence of any commercial or financial relationships that could be construed as a potential conflict of interest.

## Footnotes

1 https://portal.gdc.cancer.gov/

2 http://www.cgga.org.cn

3 http://david.ncifcrf.gov/

4 http://tide.dfci.harvard.edu/

5 https://cloud.genepattern.org/gp/

6 https://cran.r-project.org/web/packages/rms/

7 http://software.broadinstitute.org/gsea/index.jsp

8 http://www.urogene.org/cgi-bin/methprimer2/MethPrimer.cgi/

## Abbreviations

ANKRD10: ankyrin repeat domain 10
ATRX: X-linked alpha thalassemia mental retardation syndrome gene
AUC: area under the curve
BMP2: bone morphogenetic protein 2
BP: biological process
BRAF: B-Raf
BSAS: bisulfite amplicon sequencing
CC: cellular component
CDF: cumulative distribution function
CGGA: chinese glioma genome atlas
CI: confidence interval
C-index: concordance index
DAC: 5-aza-2′-deoxycytidine
DAVID: database for annotation visualization and integrated discovery
DCA: decision curve analysis
DEG: differentially expressed gene
DMSO: dimethyl sulfoxide
EGFR: epidermal growth factor receptor
ERK: extracellular signal-regulated kinase
FC: fold change
FDR: false discovery rate
GBM: glioblastoma
GO: gene ontology
GSEA: gene set enrichment analysis
HR: hazard ratio
ICB: immune checkpoint blockade
IDH: isocitrate dehydrogenase
IGP: In-group proportion
KEGG: kyoto encyclopedia of genes and genomes
K-M: kaplan-Meier
KPS: karnofsky performance score
LASSO: least absolute shrinkage and selection operator
LOXL1: lysyl oxidase Like 1
LOXL1-AS1: lysyl oxidase like 1 antisense RNA 1.
MDG: methylation-driven gene
MEM: minimum essential medium
MF: molecular function
MGMT: O6-methylgaunine-DNA-methyltransferase
OS: overall survival
qPCR: quantitative real-time polymerase chain reaction
ROC: receiver operating characteristic
RPL39L: ribosomal protein L39 Like
SubMap: subclass mapping
TCGA: the cancer genome atlas
TERT: telomerase reverse transcriptase
TIDE: tumor immune dysfunction and exclusion
TMEM52: transmembrane protein 52
TSS: transcriptional start site
VILL: villin like.
