# Supplementary figures and images for: Development and Validation of a Novel DNA Methylation-Driven Gene Based Molecular Classification and Predictive Model for Overall Survival and Immunotherapy Response in Patients With Glioblastoma: A Multiomic Analysis
Source: Front Cell Dev Biol. 2020 Sep 3;8:576996. doi: 10.3389/fcell.2020.576996 (PMC7494802; doi:10.3389/fcell.2020.576996)

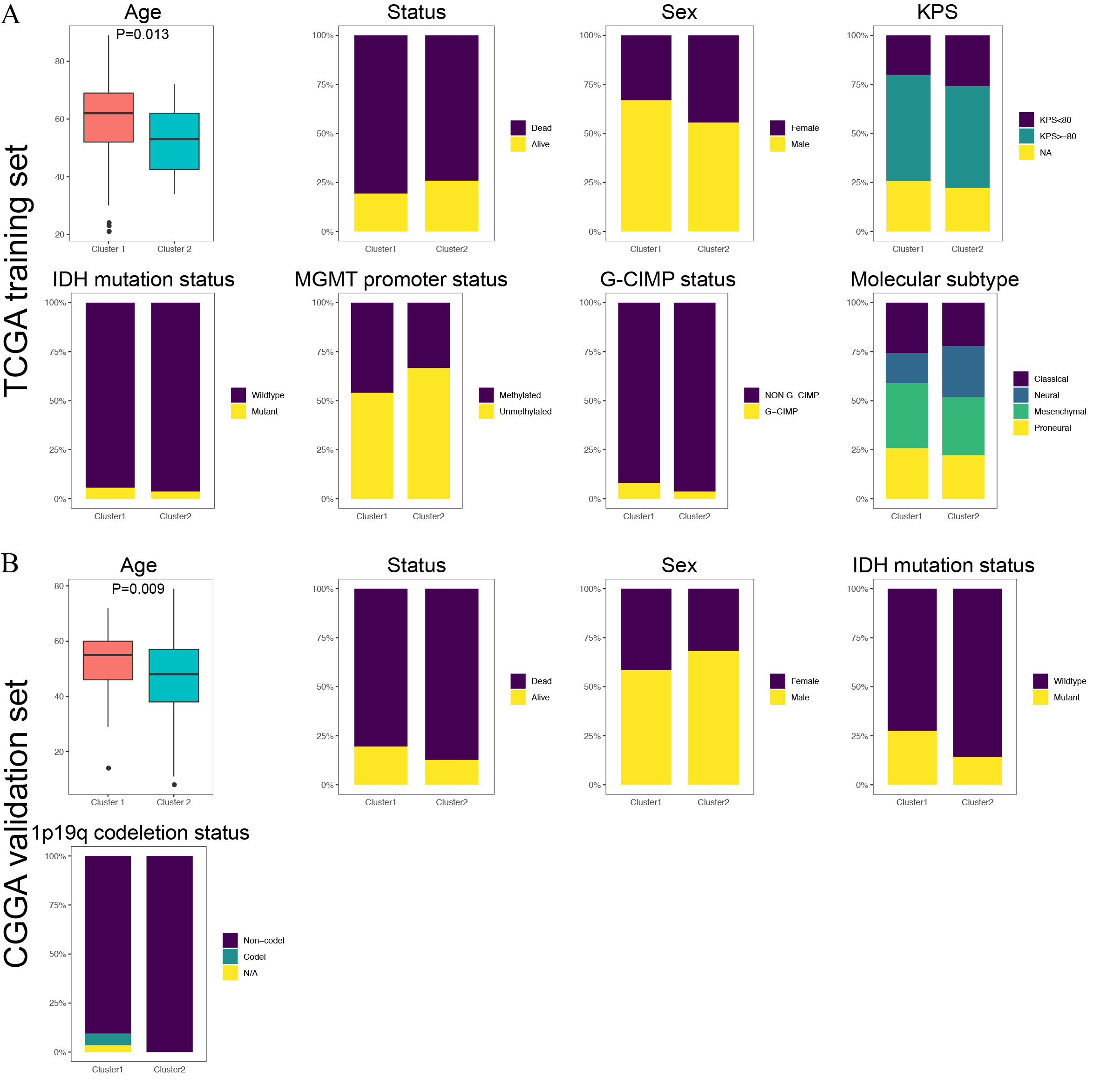

Supplement: Supplementary file 1 [file Image_1.TIF]

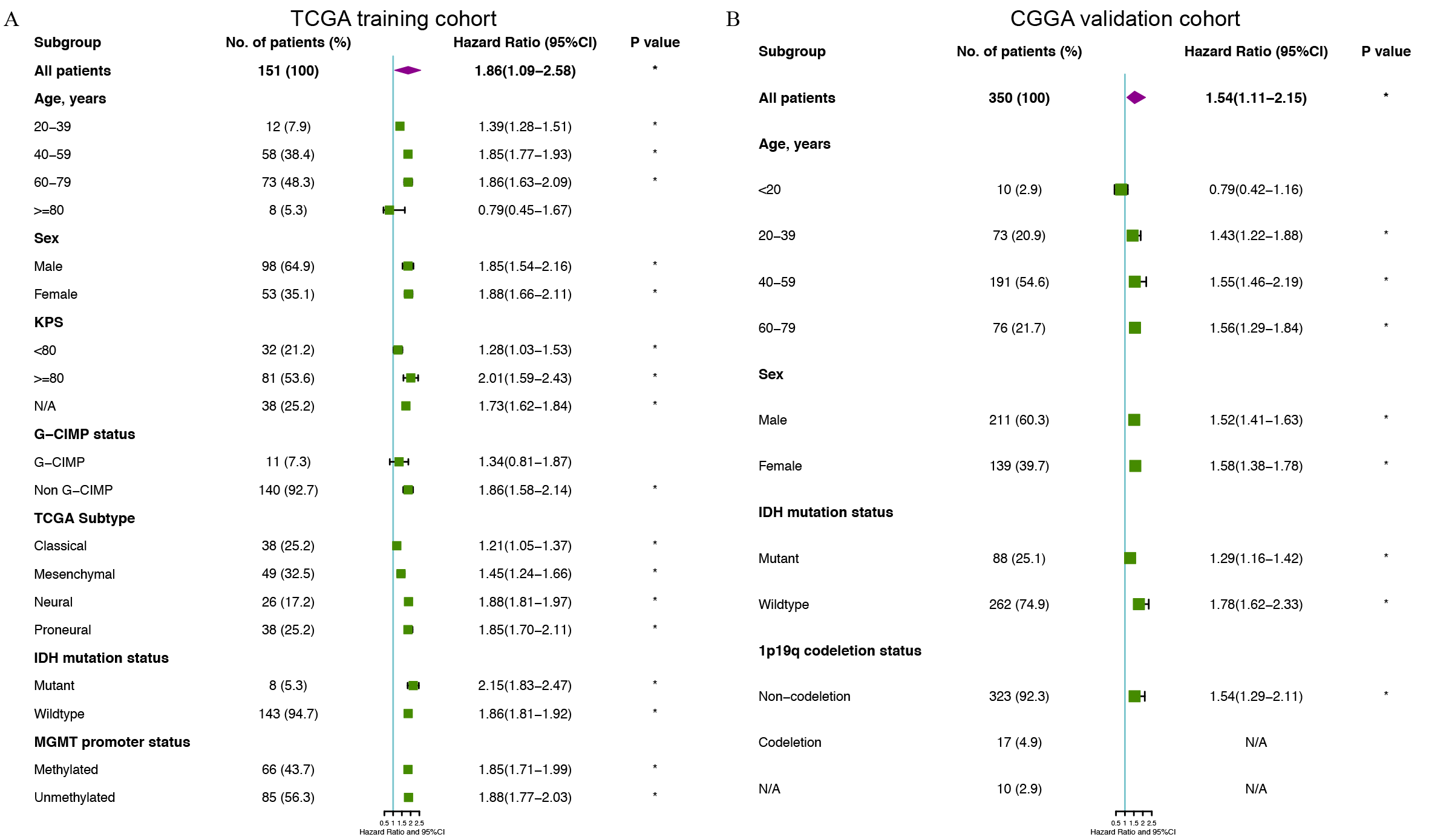

Supplement: Supplementary file 2 [file Image_2.TIF]

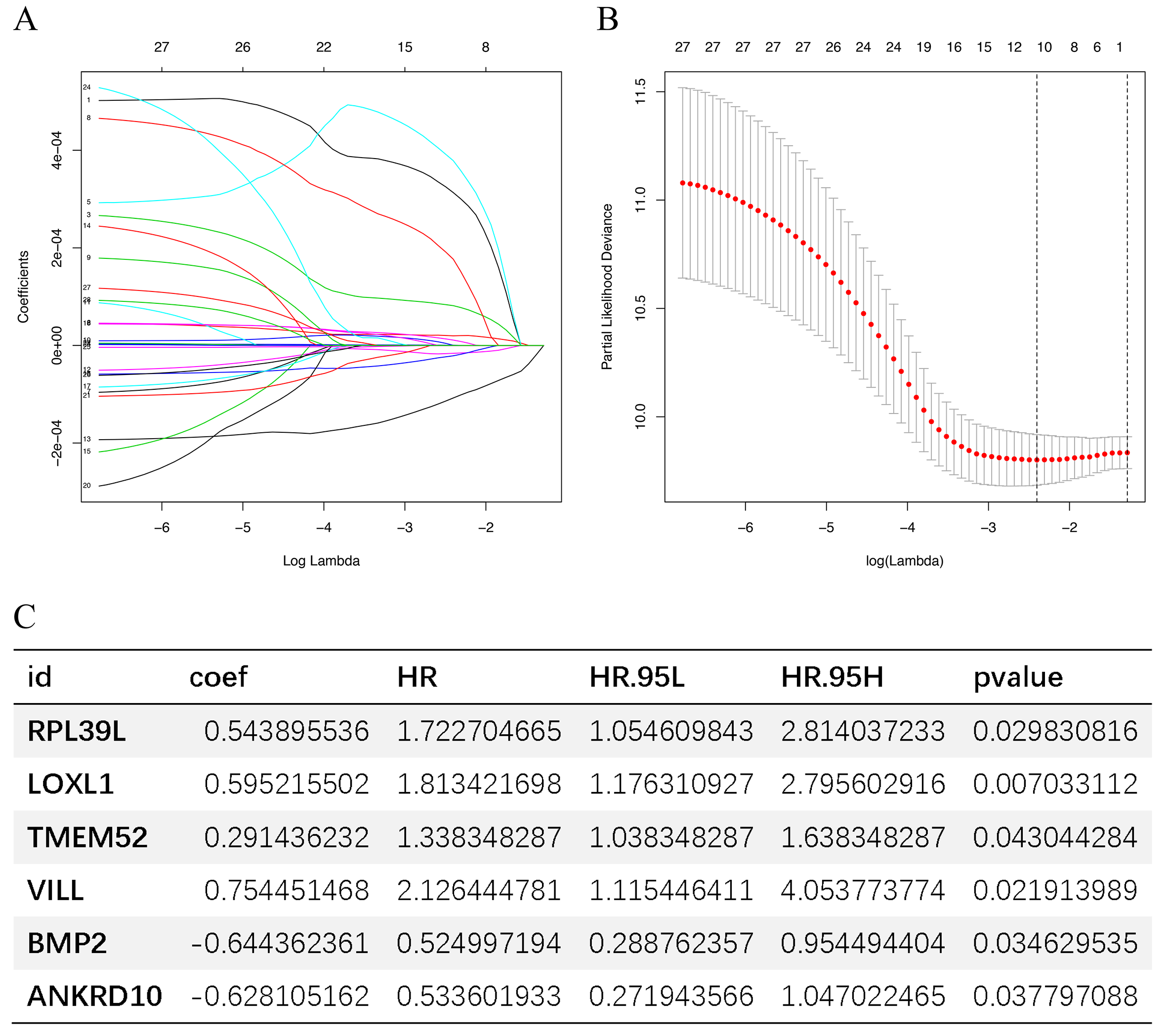

Supplement: Supplementary file 3 [file Image_3.TIF]

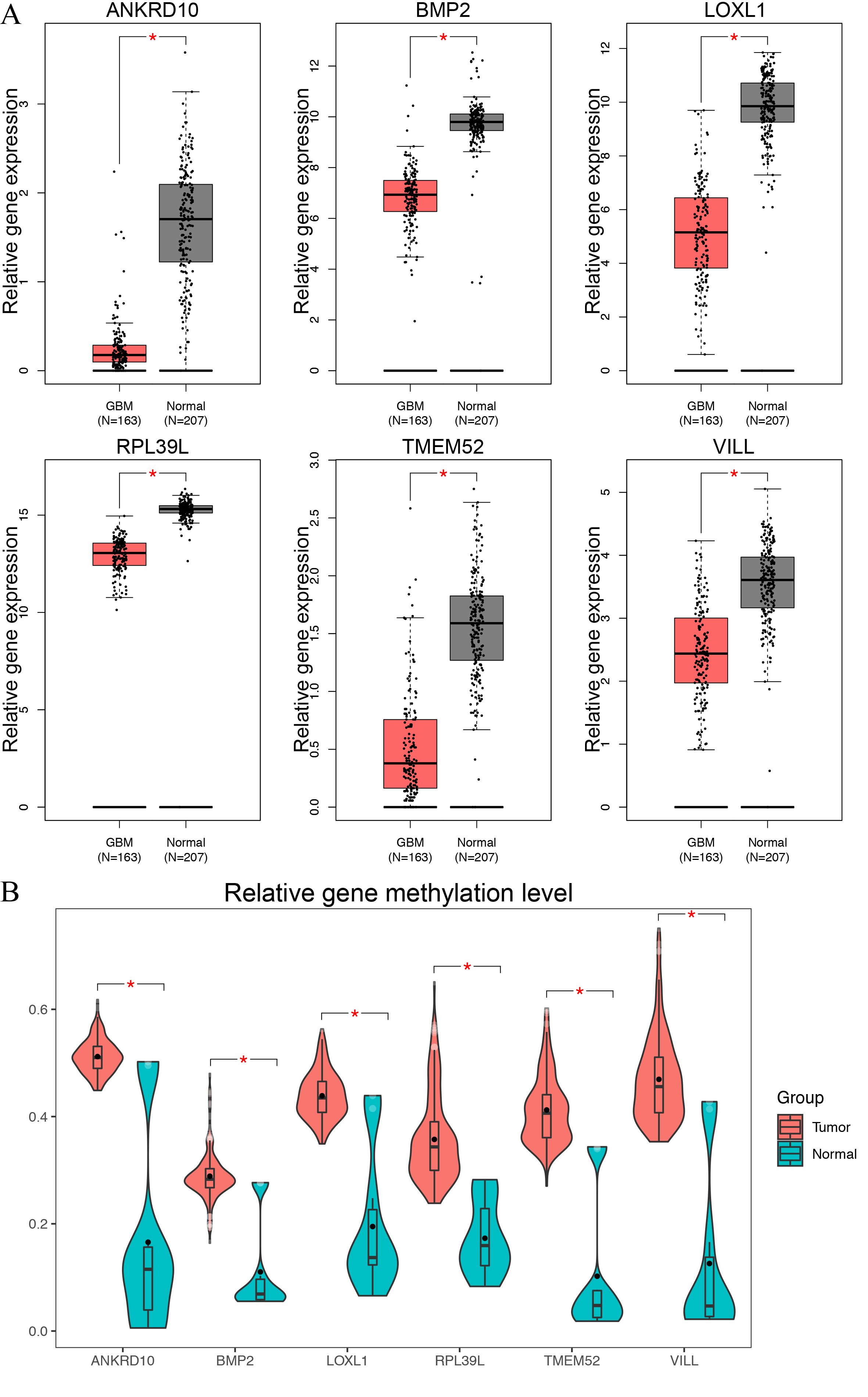

Supplement: Supplementary file 4 [file Image_4.TIF]

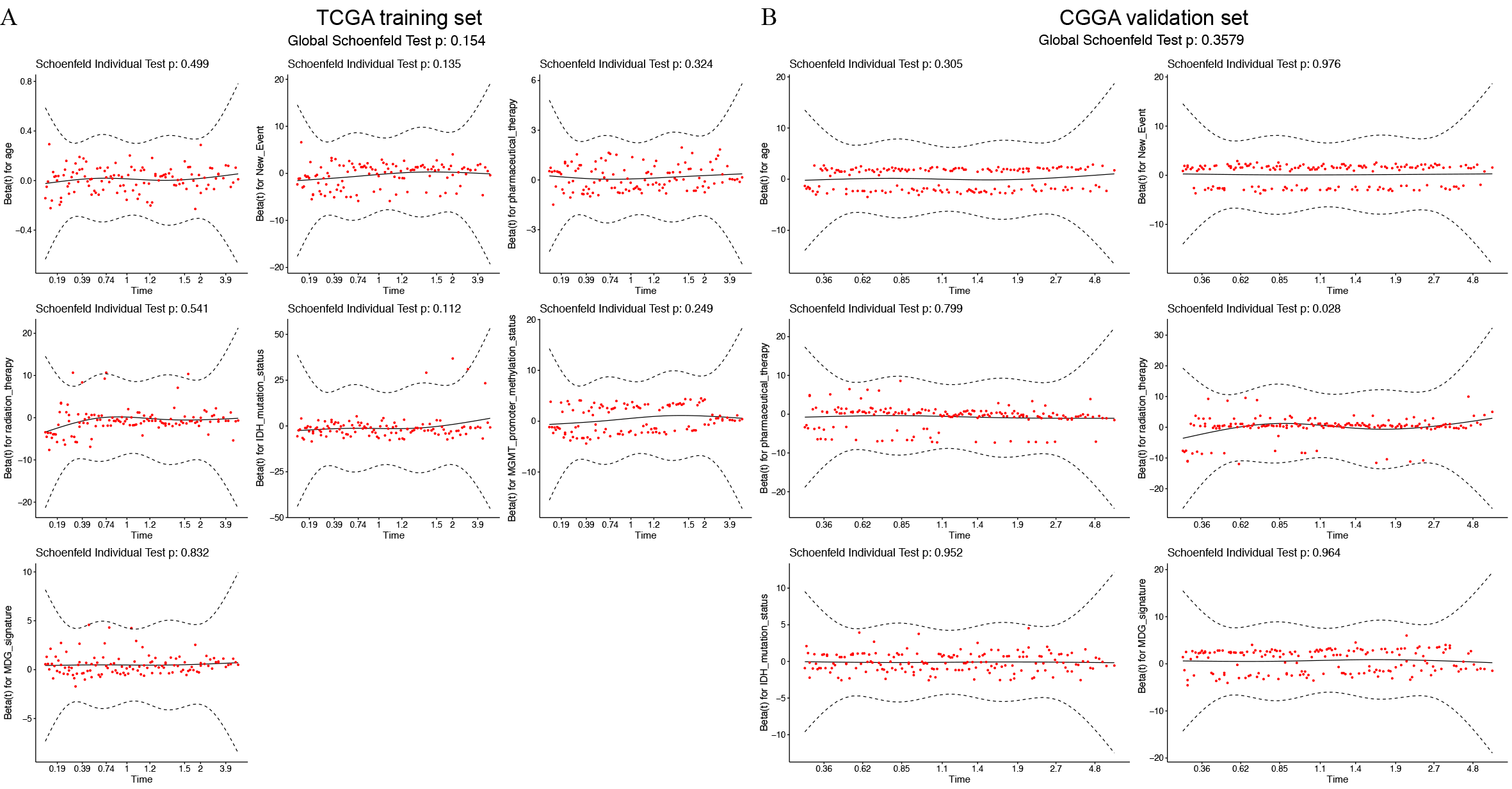

Supplement: Supplementary file 5 [file Image_5.TIF]

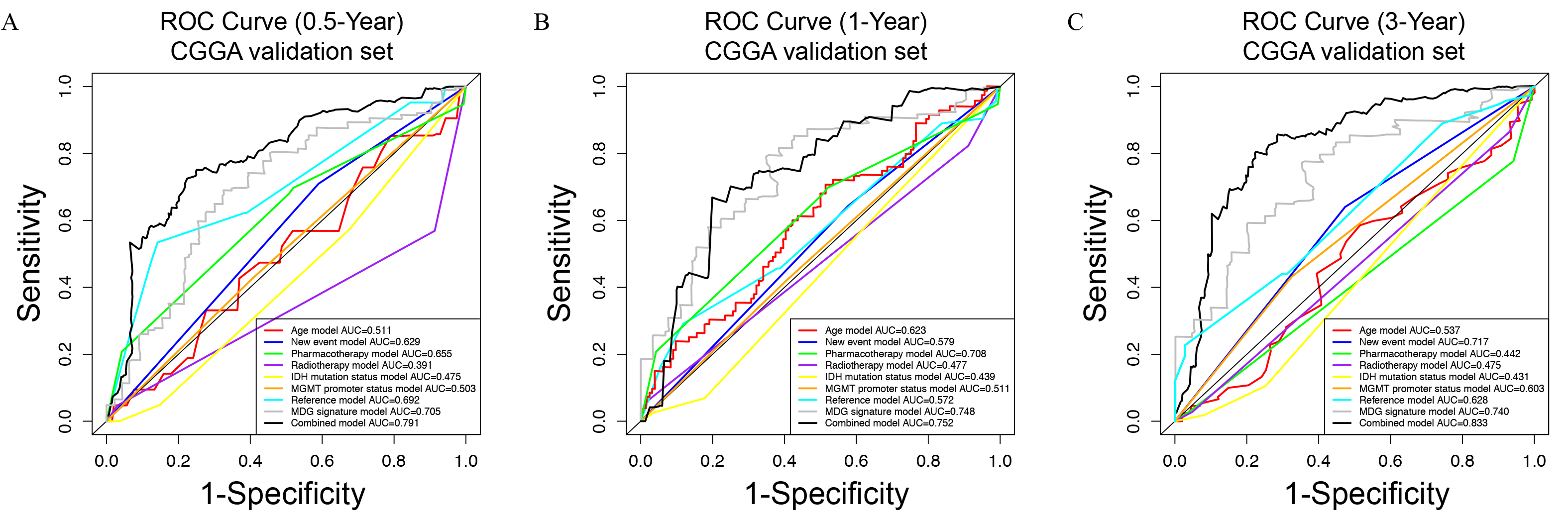

Supplement: Supplementary file 6 [file Image_6.TIF]
