# Supplementary material for: Development and Validation of a Novel DNA Methylation-Driven Gene Based Molecular Classification and Predictive Model for Overall Survival and Immunotherapy Response in Patients With Glioblastoma: A Multiomic Analysis
Source: Front Cell Dev Biol. 2020 Sep 3;8:576996. doi: 10.3389/fcell.2020.576996 (PMC7494802; doi:10.3389/fcell.2020.576996)
Supplement: Supplementary file 7 [file Table_1.DOCX]

**Supplementary Table 1.** Primers for qPCR and BSAS.

| Genes | | Forward (5’ to 3’) | Reverse (5’ to 3’) |
| --- | --- | --- | --- |
| qPCR-ANKRD10 | | CGCATTTCGGCAAGTTGGAG | TCCTGCTTGAATCAGCCAGAC |
| qPCR-BMP2 | | CATGCCATTGTTCAGACG | TGTACTAGCGACACCCACA |
| qPCR-LOXL1 | | CTGTGCTGCGGAGGAGAAG | GTAGTGGCTGAACTCGTCCA |
| qPCR-RPL39L | | CAAAATCGTCCCATCCCC | TTCTTCTCCAATGCCTCCTT |
| qPCR-TMEM52 | | CTGGTTGTGTCCGGTTCTG | TCACTGTCCATAGGGATGACTG |
| qPCR-VILL | | GCCAATGTTCGCCTGTACCAT | CGTCCCTGCCACACATAGA |
| qPCR-GAPDH | | TGACTTCAACAGCGACACCCA | CACCCTGTTGCTGTAGCCAAA |
| BSAS-ANKRD10 | **Pair 1** | CCCAGAGCGGGTGGGAGTCA | CCCCTATTTTGAATGACAGTATCACGG |
|  | **Pair 2** | GAGGGAGGAAATCCTCAGATTAC | CTTCATCAGCTGGTAACGACATTACCAGGG |
|  | **Pair 3** | ACGAGCTCAGCCGAGGAGCAAG | TGGGGTACTTGCTCTCAGAAATCTTAA |
| BSAS-BMP2 | **Pair 1** | GGGTGAGATAAATATCACAAGGCACAAAG | CACTCAATTTCCAGCCTGCTGTTTT |
|  | **Pair 2** | AGTGGGAGCCCCTGCAGGGC | CCACATGGAAAAACTCTGGTCA |
|  | **Pair 3** | AGGGTAGGGGCCTGGGACACAA | GGGGGAGGGCAAATCCCAAA |
| BSAS-LOXL1 | **Pair 1** | CATTGAACAACAGAAGGGGGTTC | CCTGGAGCCCACCTAGAGAGGAGGCAAC |
|  | **Pair 2** | CTGAATGAATAAGCAACAGGCTGGGA | ATGGGCCTCTGACTCCTGTCTGCC |
|  | **Pair 3** | ACAAAGCTAGAGCTGGGGCAAGCAAGGA | AGAGTAGCTCCCGGGTGCTG |
| BSAS-RPL39L | **Pair 1** | AATACTGACTCCAGGCAGGGAAA | GGGACCCCAAAGTCAACTTGGTGACA |
|  | **Pair 2** | TCGGTGACACGCAGACCTGAGG | CTCCTGAGTAGCTGGGGTTACAG |
|  | **Pair 3** | CTCTAAGCTAGAAACAGGGGGTG | GAGGGAGACCCTGTCTCAAAAA |
| BSAS-TMEM52 | **Pair 1** | AGTCAATGTGGCAGGAAGAAGGGCCA | CAAGAGCCTAACAGGGCCCCTGGC |
|  | **Pair 2** | GGGAAAGTGGTCTGCACCCAG | GCTTGTGGCGGACAAGCCCTC |
|  | **Pair 3** | AGCAGCCTGTGGCACGTGGGGTA | CATGGAGAGACATTGTCTGCTTTTT |
| BSAS-VILL | **Pair 1** | GGCAACAGTTCTTGGGGCAA | CCCCTAGAAGGACAGCCCACTCTGCCCC |
|  | **Pair 2** | CTCCCAGGCATGCAGGGAGG | CAGGCATAAACTCTGCCCTGGGAA |
|  | **Pair 3** | GGGAGACTGAGGCACAGGCATAAA | GGGGGCCAGACCGTGCTGCA |
