# Supplementary material for: Development and Validation of a Novel DNA Methylation-Driven Gene Based Molecular Classification and Predictive Model for Overall Survival and Immunotherapy Response in Patients With Glioblastoma: A Multiomic Analysis
Source: Front Cell Dev Biol. 2020 Sep 3;8:576996. doi: 10.3389/fcell.2020.576996 (PMC7494802; doi:10.3389/fcell.2020.576996)
Supplement: Supplementary file 8 [file Table_2.DOCX]

**Supplementary Table 2.** The relative site and the corresponding chromosomal location of CpGs located in the promoter region of the six MDGs.

| **ANKRD10** | | | | | | | | | | |
| --- | --- | --- | --- | --- | --- | --- | --- | --- | --- | --- |
| **Pair 1** | Relative CpG site | 21 | 30 | 44 | 46 | 48 | 79 | 85 | 102 | 158 |
|  | CpG site on chromosome 13 | 111568190 | 111568199 | 111568213 | 111568215 | 111568217 | 111568248 | 111568254 | 111568271 | 111568327 |
|  | Relative CpG site | 168 | 175 | 184 | 190 | 196 | 214 | 237 |  |  |
|  | CpG site on chromosome 13 | 111568337 | 111568344 | 111568353 | 111568359 | 111568365 | 111568383 | 111568406 |  |  |
| **Pair 2** | Relative CpG site | 33 | 56 | 64 | 143 | 219 | 223 | 350 |  |  |
|  | CpG site on chromosome 13 | 111565670 | 111565693 | 111565701 | 111565780 | 111565856 | 111565860 | 111565987 |  |  |
| **Pair 3** | Relative CpG site | 23 | 28 | 31 | 47 | 70 | 115 | 135 | 199 | 212 |
|  | CpG site on chromosome 13 | 111566513 | 111566508 | 111566505 | 111566489 | 111566466 | 111566421 | 111566401 | 111566337 | 111566324 |
|  | Relative CpG site | 292 | 312 |  |  |  |  |  |  |  |
|  | CpG site on chromosome 13 | 111566244 | 111566224 |  |  |  |  |  |  |  |
| **BMP2** | | | | | | | | | | |
| **Pair 1** | Relative CpG site | 86 | 95 | 111 | 156 | 171 | 266 |  |  |  |
|  | CpG site on chromosome 20 | 6747540 | 6747549 | 6747565 | 6747610 | 6747625 | 6747720 |  |  |  |
| **Pair 2** | Relative CpG site | 23 | 30 | 68 | 86 | 128 | 140 | 142 | 163 | 170 |
|  | CpG site on chromosome 20 | 6749652 | 6749645 | 6749607 | 6749589 | 6749547 | 6749535 | 6749533 | 6749512 | 6749505 |
|  | Relative CpG site | 183 | 185 | 193 | 208 |  |  |  |  |  |
|  | CpG site on chromosome 20 | 6749492 | 6749490 | 6749482 | 6749467 |  |  |  |  |  |
| **Pair 3** | Relative CpG site | 186 | 198 | 221 | 240 | 260 | 276 | 311 |  |  |
|  | CpG site on chromosome 20 | 6750365 | 6750377 | 6750400 | 6750419 | 6750439 | 6750455 | 6750490 |  |  |
| **LOXL1** | | | | | | | | | | |
| **Pair 1** | Relative CpG site | 24 | 89 | 196 | 266 | 281 |  |  |  |  |
|  | CpG site on chromosome 15 | 74217906 | 74217841 | 74217734 | 74217664 | 74217649 |  |  |  |  |
| **Pair 2** | Relative CpG site | 28 | 31 | 41 | 54 | 73 | 75 | 90 | 93 | 108 |
|  | CpG site on chromosome 15 | 74218782 | 74218779 | 74218769 | 74218756 | 74218737 | 74218735 | 74218720 | 74218717 | 74218702 |
|  | Relative CpG site | 111 | 188 | 221 | 254 | 279 | 340 |  |  |  |
|  | CpG site on chromosome 15 | 74218699 | 74218622 | 74218589 | 74218556 | 74218531 | 74218470 |  |  |  |
| **Pair 3** | Relative CpG site | 42 | 48 | 64 | 136 | 145 | 171 | 183 | 195 | 206 |
|  | CpG site on chromosome 15 | 74219043 | 74219049 | 74219065 | 74219137 | 74219146 | 74219172 | 74219184 | 74219196 | 74219207 |
|  | Relative CpG site | 213 | 224 | 231 | 235 | 241 | 267 | 293 | 306 | 311 |
|  | CpG site on chromosome 15 | 74219214 | 74219225 | 74219232 | 74219236 | 74219242 | 74219268 | 74219294 | 74219307 | 74219312 |
|  | Relative CpG site | 315 | 325 | 330 |  |  |  |  |  |  |
|  | CpG site on chromosome 15 | 74219316 | 74219326 | 74219331 |  |  |  |  |  |  |
| **RPL39L** | | | | | | | | | | |
| **Pair 1** | Relative CpG site | 42 | 84 | 87 | 95 | 110 | 129 | 141 | 149 | 151 |
|  | CpG site on chromosome 3 | 186857702 | 186857660 | 186857657 | 186857649 | 186857634 | 186857615 | 186857603 | 186857595 | 186857593 |
|  | Relative CpG site | 180 | 188 | 192 | 205 | 226 | 243 | 267 | 269 |  |
|  | CpG site on chromosome 3 | 186857564 | 186857556 | 186857552 | 186857539 | 186857518 | 186857501 | 186857477 | 186857475 |  |
| **Pair 2** | Relative CpG site | 33 | 42 | 46 | 48 | 68 | 77 | 110 | 113 | 138 |
|  | CpG site on chromosome 3 | 186857083 | 186857074 | 186857070 | 186857068 | 186857048 | 186857039 | 186857006 | 186857003 | 186856978 |
|  | Relative CpG site | 141 | 151 | 156 | 163 | 172 | 175 | 186 | 218 | 236 |
|  | CpG site on chromosome 3 | 186856975 | 186856965 | 186856960 | 186856953 | 186856944 | 186856941 | 186856930 | 186856898 | 186856880 |
|  | Relative CpG site | 287 | 290 | 320 | 337 | 343 | 353 | 362 |  |  |
|  | CpG site on chromosome 3 | 186856829 | 186856826 | 186856796 | 186856779 | 186856773 | 186856763 | 186856754 |  |  |
| **Pair 3** | Relative CpG site | 73 | 101 | 106 | 110 | 125 | 133 | 163 | 191 | 203 |
|  | CpG site on chromosome 3 | 186856585 | 186856613 | 186856618 | 186856622 | 186856637 | 186856645 | 186856675 | 186856703 | 186856715 |
|  | Relative CpG site | 205 | 207 | 240 | 249 | 259 | 265 | 282 | 312 | 315 |
|  | CpG site on chromosome 3 | 186856717 | 186856719 | 186856752 | 186856761 | 186856771 | 186856777 | 186856794 | 186856824 | 186856827 |
| **TMEM52** | | | | | | | | | | |
| **Pair 1** | Relative CpG site | 69 | 166 | 230 | 340 | 359 |  |  |  |  |
|  | CpG site on chromosome 1 | 1852073 | 1851976 | 1851912 | 1851802 | 1851783 |  |  |  |  |
| **Pair 2** | Relative CpG site | 22 | 81 | 90 | 125 | 148 | 174 | 180 | 192 | 217 |
|  | CpG site on chromosome 1 | 1851543 | 1851484 | 1851475 | 1851440 | 1851417 | 1851391 | 1851385 | 1851373 | 1851348 |
|  | Relative CpG site | 237 | 247 | 254 | 281 | 285 | 289 | 300 | 303 | 309 |
|  | CpG site on chromosome 1 | 1851328 | 1851318 | 1851311 | 1851284 | 1851280 | 1851276 | 1851265 | 1851262 | 1851256 |
|  | Relative CpG site | 320 | 330 | 333 | 340 | 363 |  |  |  |  |
|  | CpG site on chromosome 1 | 1851245 | 1851235 | 1851232 | 1851225 | 1851202 |  |  |  |  |
| **Pair 3** | Relative CpG site | 33 | 35 | 38 | 44 | 48 | 52 | 55 | 66 | 76 |
|  | CpG site on chromosome 1 | 1850319 | 1850317 | 1850314 | 1850308 | 1850304 | 1850300 | 1850297 | 1850286 | 1850276 |
|  | Relative CpG site | 88 | 112 | 118 | 124 | 134 | 138 | 144 | 189 | 200 |
|  | CpG site on chromosome 1 | 1850264 | 1850240 | 1850234 | 1850228 | 1850218 | 1850214 | 1850208 | 1850163 | 1850152 |
|  | Relative CpG site | 202 | 215 | 221 | 240 | 252 | 324 | 343 | 346 | 353 |
|  | CpG site on chromosome 1 | 1850150 | 1850137 | 1850131 | 1850112 | 1850100 | 1850028 | 1850009 | 1850006 | 1849999 |
| **VILL** | | | | | | | | | | |
| **Pair 1** | Relative CpG site | 31 | 35 | 71 | 128 | 241 | 263 |  |  |  |
|  | CpG site on chromosome 3 | 38033934 | 38033938 | 38033974 | 38034031 | 38034144 | 38034166 |  |  |  |
| **Pair 2** | Relative CpG site | 53 | 86 | 114 | 130 | 153 | 211 | 224 | 231 | 243 |
|  | CpG site on chromosome 3 | 38035234 | 38035267 | 38035295 | 38035311 | 38035334 | 38035392 | 38035405 | 38035412 | 38035424 |
|  | Relative CpG site | 282 | 289 | 326 |  |  |  |  |  |  |
|  | CpG site on chromosome 3 | 38035463 | 38035470 | 38035507 |  |  |  |  |  |  |
| **Pair 3** | Relative CpG site | 40 | 55 | 80 | 87 | 128 | 130 | 147 | 188 | 196 |
|  | CpG site on chromosome 3 | 38035554 | 38035569 | 38035594 | 38035601 | 38035642 | 38035644 | 38035661 | 38035702 | 38035710 |
|  | Relative CpG site | 205 | 228 | 251 | 260 | 269 | 276 | 294 | 305 | 311 |
|  | CpG site on chromosome 3 | 38035719 | 38035742 | 38035765 | 38035774 | 38035783 | 38035790 | 38035808 | 38035819 | 38035825 |
|  | Relative CpG site | 317 | 324 | 329 | 346 | 357 |  |  |  |  |
|  | CpG site on chromosome 3 | 38035831 | 38035838 | 38035843 | 38035860 | 38035871 |  |  |  |  |
