# Supplementary material for: Development and Validation of a Novel DNA Methylation-Driven Gene Based Molecular Classification and Predictive Model for Overall Survival and Immunotherapy Response in Patients With Glioblastoma: A Multiomic Analysis
Source: Front Cell Dev Biol. 2020 Sep 3;8:576996. doi: 10.3389/fcell.2020.576996 (PMC7494802; doi:10.3389/fcell.2020.576996)
Supplement: Supplementary file 10 [file Table_4.DOCX]

**Supplementary Table 4.** The multivariate Cox analysis of the 4 groups based on the methylations and expressions of the six MDGs.

| Genes | Methylation and expression of MDGs | HR | 95% CI | P value |
| --- | --- | --- | --- | --- |
| ANKRD10 | High Meth/Low Exp | Reference |  |  |
|  | High Meth/High Exp | 0.57 | 0.30-1.12 | 0.10 |
|  | Low Meth/Low Exp | 0.33 | 0.14-0.77 | **0.01** |
|  | Low Meth/High Exp | 0.30 | 0.12-0.74 | **0.01** |
| BMP2 | High Meth/Low Exp | Reference |  |  |
|  | High Meth/High Exp | 0.96 | 0.47-1.97 | 0.91 |
|  | Low Meth/Low Exp | 1.96 | 0.83-4.66 | 0.13 |
|  | Low Meth/High Exp | 0.82 | 0.63-0.98 | **0.04** |
| LOXL1 | High Meth/Low Exp | Reference |  |  |
|  | High Meth/High Exp | 2.01 | 0.99-4.06 | 0.05 |
|  | Low Meth/Low Exp | 2.46 | 1.11-5.43 | **0.03** |
|  | Low Meth/High Exp | 2.52 | 1.67-3.45 | **0.03** |
| RPL39L | High Meth/Low Exp | Reference |  |  |
|  | High Meth/High Exp | 0.88 | 0.32-2.42 | 0.79 |
|  | Low Meth/Low Exp | 0.65 | 0.25-1.70 | 0.38 |
|  | Low Meth/High Exp | 2.59 | 1.35-4.97 | **0.004** |
| TMEM52 | High Meth/Low Exp | Reference |  |  |
|  | High Meth/High Exp | 0.96 | 0.44-2.11 | 0.93 |
|  | Low Meth/Low Exp | 1.41 | 0.63-3.13 | 0.41 |
|  | Low Meth/High Exp | 1.94 | 1.47-2.89 | **0.04** |
| VILL | High Meth/Low Exp | Reference |  |  |
|  | High Meth/High Exp | 1.73 | 0.72-4.17 | 0.22 |
|  | Low Meth/Low Exp | 1.16 | 0.51-2.67 | 0.72 |
|  | Low Meth/High Exp | 1.17 | 1.05-1.31 | **0.02** |

**Abbreviations:** HR, hazard ratio; 95% CI, 95% confidence interval.

Bold type of P value means P < 0.05.
