# Supplementary material for: Development and Validation of a Novel DNA Methylation-Driven Gene Based Molecular Classification and Predictive Model for Overall Survival and Immunotherapy Response in Patients With Glioblastoma: A Multiomic Analysis
Source: Front Cell Dev Biol. 2020 Sep 3;8:576996. doi: 10.3389/fcell.2020.576996 (PMC7494802; doi:10.3389/fcell.2020.576996)
Supplement: Supplementary file 11 [file Data_Sheet_1.docx]

**Supplementary Figure Legends**

**Supplementary Figure 1.** The comparisons of the clinicopathological variables between the two clusters of GBM patients in the TCGA (**A**) and CGGA cohorts (**B**).

**Supplementary Figure 2.** The subgroup analysis of GBM patients in the training **(A)** and validation **(B)** cohort. The purple diamond represented that Cluster 1 patients showed worse OS than Cluster 2, with HR > 1. * means P < 0.05.

**Supplementary Figure 3.** The prognosis-related MDGs were screened by the LASSO and multivariate Cox regression analysis. (**A**) The coefficient profile plot was produced against the log(lambda) sequence. Vertical line was drawn at the value selected using ten-fold cross-validation, where optimal lambda resulted in ten features with nonzero coefficients. (**B**) Optimal parameter (lambda) selection in the LASSO model used ten-fold cross-validation via minimum criteria. The partial likelihood deviance (binomial deviance) curve was plotted versus log(lambda). Dotted vertical lines were drawn at the optimal values by using the minimum criteria and the I standard error of the minimum criteria. (**C**) Following LASSO regression analysis, the 6 MDGs identified by multivariate Cox regression analysis.

**Supplementary Figure 4.** The expression and methylation levels of the 6 MDGs. (**A**) The expression levels of the 6 MDGs in 163 GBM samples and 207 normal cerebral samples. (**B**) The methylation levels of the 6 MDGs in GBM and normal cerebral tissues were visualized by violin plots. Asterisks means p < 0.05.

**Supplementary Figure 5.** The proportional hazard (PH) assumption of the Cox regression analysis for the clinical variables and combined model in TCGA (A) and CGGA (B) cohort via the Schoenfeld Residuals Test.

**Supplementary Figure 6.** The prognostic performances of the nomogram demonstrated by the ROC curve for predicting the 0.5-, 1‐, and 3‐year OS rate in CGGA validation cohort.
